# Case Report: Pyogenic Arthritis, Pyoderma Gangrenosum, and Acne: A Single-Center Experience and Literature Review

**DOI:** 10.3389/fimmu.2021.735851

**Published:** 2021-10-22

**Authors:** Yumei Wang, Na Wu, Keyi Yu, Min Shen

**Affiliations:** ^1^ Department of Rheumatology and Clinical Immunology, Chinese Academy of Medical Sciences and Peking Union Medical College, National Clinical Research Center for Dermatologic and Immunologic Diseases (NCRC-DID), Ministry of Science and Technology, State Key Laboratory of Complex Severe and Rare Diseases, Peking Union Medical College Hospital (PUMCH), Key Laboratory of Rheumatology and Clinical Immunology, Ministry of Education, Beijing, China; ^2^ Department of Rheumatology, Aerospace Center Hospital, Beijing, China; ^3^ Department of Orthopaedic Surgery, State Key Laboratory of Complex Severe and Rare Diseases, Peking Union Medical College Hospital, Chinese Academy of Medical Sciences and Peking Union Medical College, Beijing, China

**Keywords:** autoinflammatory diseases, PAPA syndrome, PSTPIP1, pyoderma gangrenosum, interleukin-1

## Abstract

**Objectives:**

This study aims to describe the characteristics of patients diagnosed with pyogenic arthritis, pyoderma gangrenosum, and acne (PAPA) syndrome at a single center in China and provide an up-to-date literature review.

**Methods:**

The clinical data and genotype of three Chinese Han patients were carefully documented and studied. We also conducted a systematic literature review on PAPA syndrome.

**Results:**

A total of three patients were diagnosed with PAPA syndrome at our center from 2018 to 2020. Arthritis was observed in all three patients, while pyoderma gangrenosum (PG) was found in two patients and acne in one patient. Other manifestations included pathergy reaction, intermittent fever, oral ulcer, keratitis, proteinuria, and hematuria. The *PSTPIP1* A230T mutation was identified in two patients, and a novel Y119C variation was revealed in a sporadic patient. A total of 76 patients with PAPA syndrome reported in 29 articles were included in our literature review. The classical triad of arthritis, PG, and acne was visible in only 16 (25.4%) patients, while 24 (38.1%) exhibited only one major symptom. Skin lesions were more commonly seen in patients with adult-onset disease than those with childhood-onset disease (100 *vs*. 83%), whereas arthritis was less common (50 *vs*. 98.1%). Steroid and/or biological agents were effective in most patients.

**Conclusions:**

The rarity and phenotypic heterogeneity associated with PAPA syndrome make the diagnosis a huge challenge to physicians, especially in adult patients. A significant portion of patients did not exhibit the full spectrum of the classical triad. Accordingly, gene testing is critically helpful for diagnosis.

## Introduction

Pyogenic arthritis, pyoderma gangrenosum, and acne (PAPA) syndrome (OMIM #604416) is an autosomal dominant inherited disorder. It is a rare autoinflammatory disease mediated by interleukin (IL)-1β that is overproduced by either “inflammasome” or “pyroptosome” mechanisms in the presence of PAPA causal mutations (1). Since pyoderma and cystic acne may not occur in the early disease course, PAPA syndrome is prone to be misdiagnosed as a joint abscess. Unlike septic arthritis, PAPA syndrome is characterized by recurrent joint symptoms after surgical treatment and sterile drainage of synovial fluid. Its diagnosis depends on gene sequencing. No consensus has been reached on a standard treatment for strategy for PAPA syndrome owing to the rarity of this disease, although tumor necrosis factor α (TNFα) inhibitors and IL-1 antagonists have been reported to be effective in a few cases.

PAPA syndrome has been sparsely documented in the Chinese population in English literature (2). Herein we reported three adult patients with PAPA syndrome from two unrelated families in China and conducted a review of the literature.

## Patients and Methods

A total of three patients with PAPA syndrome were diagnosed and followed up in our tertiary medical center from 2018 to 2020. The complete medical records and laboratory data were collected. Whole-exome sequencing by next-generation sequencing was performed in the Center for Genetic Testing, Joy Orient Translational Medicine Research Centre Co., Ltd., Beijing, China.

A literature search in PubMed was performed using the terms “PAPA syndrome” and “*PSTPIP1* gene mutation” from 1997 to 2020. The eligibility criteria for inclusion were English language publications with no restrictions on age, sex, or ethnicity. We also screened the references in the selected articles. Finally, 29 articles containing a total of 76 patients with PAPA syndrome were included and reviewed.

This study was approved by the Institutional Review Board of Peking Union Medical College Hospital and performed according to the Declaration of Helsinki. Informed consents were obtained from all participants.

## Results

### Patient 1

**Figure 1:**
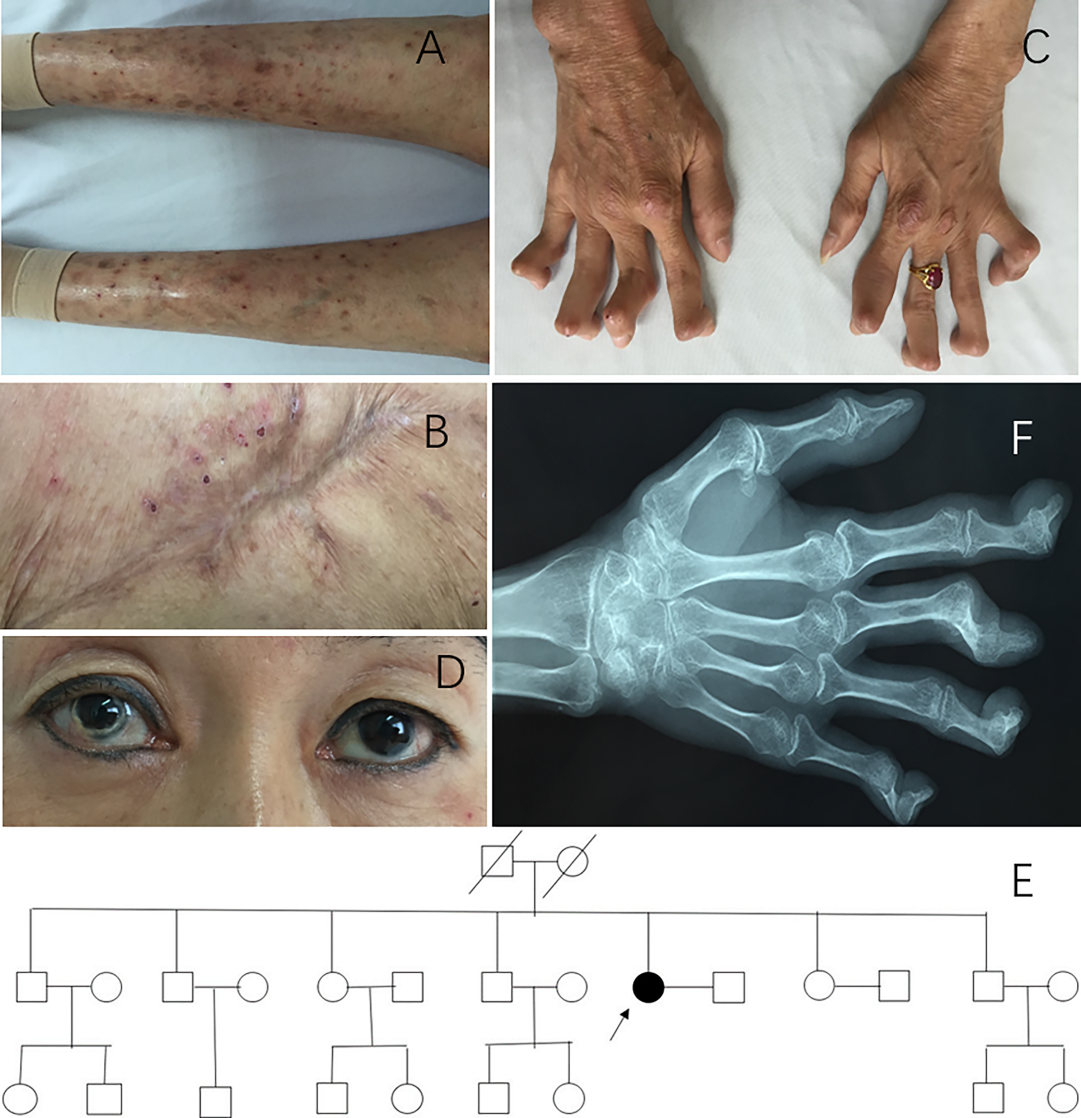
Clinical manifestations of patient 1. **(A)** Blisters and scars on lower limbs. **(B)** Healed pyoderma gangrenosum on the buttocks. **(C)** Hand deformity. **(D)** Corneal deformation could be observed on physical examination. **(E)** Pedigree of patient 1. Arrow, proband; black symbols, affected individuals; open symbols, unaffected individuals. **(F)** X-ray of hands showing osteoporosis, multiple joint space narrowing, hyperosteogeny, subluxation, and contracture.

A 49-year-old Chinese Han woman presented with rashes for 37 years and recurrent joint swelling and pain for 31 years. Since the age of 12, the patient had developed blister-like rashes without any inducement, which initially appeared on her lower limbs and buttocks and then spread to her upper limbs and trunk. After scratching, the blisters spilled and scabbed (**Figure 1A**). When she was over 30 years old, there was a large area of ulceration in the buttock, and then it healed (**Figure 1B**). Skin biopsy at that time revealed a large number of neutrophil infiltration in the superficial and middle dermis and also lymphocytes infiltration, which conformed to pyoderma gangrenosum (PG). At the age of 18, the patient developed polyarthritis which caused irreversible joint damage and deformity (**Figure 1C**). During the past 10 years, she experienced recurrent red eyes and loss of vision in both eyes and was diagnosed with keratitis. Corneal deformation was observed on physical examination (**Figure 1D**). She also complained of low fever, weight loss (with a body weight of only 35 kg), and headache in the past year. The patient had no acne, oral ulcer, chest pain, abdominal pain, periorbital edema, hearing loss, or lymph node enlargement. She did not take any medicine before she visited our clinic. She previously experienced 10 second-trimester miscarriages. She denied a family history of autoinflammatory diseases (**Figure 1E**). Her parents died of liver cirrhosis and cerebellar atrophy, respectively.

The complete blood count (CBC) and complete biochemistry panel were within the normal range. The urine analysis showed positive occult blood and trace protein. Serological testing for autoantibodies was negative. The erythrocyte sedimentation rate (ESR) was 48 mm/h (normal range 0–20 mm/h), and C-reactive protein (CRP) was 8.72 mg/L (normal range 0–3 mg/L). An X-ray of her hands showed osteoporosis, multiple joint space narrowing, hyperosteogeny, subluxation, and contracture (**Figure 1F**). Gene testing identified a heterozygous c.356A>G, p.Tyr119Cys (p.Y119C) variation in the *PSTPIP1* gene (NM_003978). She was eventually diagnosed with PAPA syndrome.

Due to financial issues, she refused treatment with biological agents. Accordingly, she was treated with prednisone therapy at 20 mg per day, combined with methotrexate at 10 mg per week, and cyclosporine at 50 mg twice a day. Her rash, arthritis, and ophthalmitis significantly improved, and the acute phase reactants ESR and CRP decreased to normal range. The symptoms remained stable at 1-year follow-up, and prednisone was tapered to 5 mg daily.

### Patient 2

**Figure 2:**
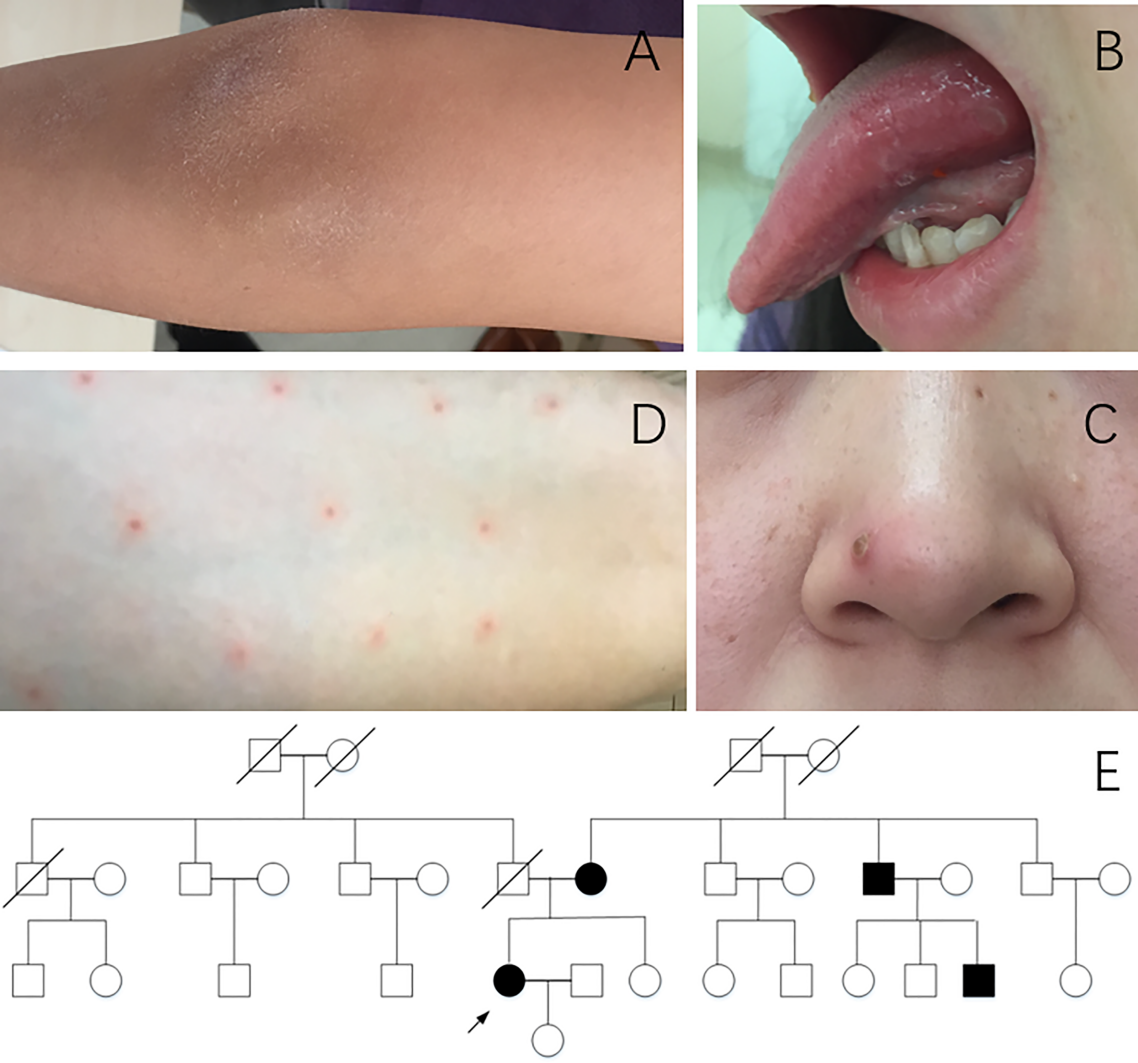
Clinical manifestations of patient 2. **(A)** Arthritis of the right elbow. **(B)** Oral ulcer. **(C)** Acne. **(D)** Pathergy reaction. **(E)** Pedigree of patient 2. Arrow, proband; black symbols, affected individuals; open symbols; unaffected individuals.

The patient was a 34-year-old Chinese Han woman who complained of recurrent arthritis for nearly 10 years. Her right wrist, bilateral knees, and right elbow were successively involved (**Figure 2A**), approximately once every 1 to 2 years. She was only treated with short-term prednisone, combined with sulphasalazine or methotrexate during flares, which improved her arthritis. Arthroscopy of the right knee was once performed, and the pathology suggested chronic synovitis. She also suffered from recurrent oral ulcers more than three times a year (**Figure 2B**), acne (**Figure 2C**), and poor wound healing. At the age of 31, she presented with huge genital ulcers and was diagnosed with PG after a pathological examination. No documentation of the treatment given at another local hospital was available; however, the treatment was effective, with no PG-related symptoms since then. Pathergy reaction had also been noticed in recent years when she underwent acupuncture (**Figure 2D**). She had no fever, chest pain, history of thrombosis, gastrointestinal tract symptoms, hearing loss, or red eyes. The pedigree analysis (**Figure 2E**) revealed that her mother had recurrent arthritis and acne, and arthroscopy confirmed synovitis. Her uncle had polyarthritis since childhood, and one of her cousins also complained of recurrent arthritis accompanied by fever.

Hypochromic microcytic anemia (Hb 86g/L) and elevated ESR (59 mm/h) and CRP (148.9 mg/L) were noted in laboratory testing. The CBC, urine analysis, and complete biochemistry panel were normal. Testing for autoantibodies was negative. The magnetic resonance imaging of knee joints showed joint effusion, diffuse synovial thickening, bone marrow edema, and surrounding soft tissue swelling. Gene testing demonstrated a heterozygous c.688G>A, p.Ala230Thr (p.A230T) variation of *PSTPIP1* in the four affected kindreds, but not in other healthy members of this family.

Given the low frequency of arthritis attacks and the mild severity of symptoms, she was treated with leflunomide and diclofenac at the onset of arthritis. When the symptoms improved, the medications were discontinued.

### Patient 3

Patient 3 was a 26-year-old Chinese Han man that happened to be the cousin of patient 2. The patient developed recurrent arthritis and fever at the age of 2 years old. His episodes of arthritis lasted several weeks during each flare, involving bilateral knees, ankles, and elbows, accompanied by a low-grade fever. There were fatigue and weight loss, but no acne and oral or genital ulcers. Laboratory studies showed elevated acute phase reactants (ESR 56 mm/h and CRP 37 mg/L); however, the blood and joint fluid cultures were negative. The antinuclear antibody was positive (1:160). The patient underwent arthroscopic examination, and the pathology indicated synovitis. He had a good response to low-dose prednisone therapy without disease-modifying antirheumatic drugs. The etiology for his arthritis and fever was unknown until his cousin (patient 2) was diagnosed with PAPA syndrome, and the same *PSTPIP1* A230T variation was found. He was also diagnosed with PAPA syndrome due to his childhood-onset, typical symptom of recurrent pyogenic arthritis, combined with the family history of PAPA and *PSTPIP1* A230T variation. Adalimumab was recommended for the treatment of his arthritis. Since he had no flares of arthritis in the past year, he had not received the medicine. He was still monitored by regular follow-ups.

### Literature Review

PAPA syndrome, first described in 1997, is an exceedingly rare autoinflammatory disease (3). In 2002, the gene proline⁄serine⁄threonine phosphatase-interacting protein 1 (*PSTPIP1*) on chromosome 15q24-25.1 was identified to play a major role in this disease (4). PAPA syndrome manifests as pyogenic sterile arthritis, PG, and cystic acne and is usually associated with *PSTPIP1* gene mutations. Other autoinflammatory syndromes caused by mutations in the *PSTPIP1* gene, such as PAMI, PASH, PAPASH, PsAPASH, PAC, or PASS syndrome, have recently been included in the group of PAPA spectrum disorders (5–7).

Mutations in the *PSTPIP1* gene influence the ability of PSTPIP1 to phosphorylate proinflammatory pyrin domains, leading to the accumulation, activation, and reduced inhibition of the inflammasome, release of IL-1β and IL-18, activation of caspase 1, and dysregulated downstream TNF-α production in mononuclear cells (8). Mistry P et al. expounded the importance of neutrophil extracellular traps in the pathogenesis of PAPA and further confirmed the role of IL-1 signaling in exacerbated neutrophil responses (9). Moreover, PSTPIP1 has been reported to be highly expressed in T cells, implying improper activation of the adaptive immune response in PAPA syndrome patients (10). Furthermore, some patients have been diagnosed based on clinical manifestations with negative genetic tests. Hence, further studies are required to explore the pathogenesis of the disease.

The demographic data, clinical phenotypes, and laboratory features of these three patients are summarized in **Table 1**. We also conducted an English literature review of PAPA syndrome. A total of 76 patients with detailed information were included. Among them, there were 53 men (69.7%) and 23 women (30.3%). The age of onset was from infancy to 28 years, with a proportion of adult onset, 6.6% (*n* = 5), and childhood onset, 93.4% (*n* = 71). The initial manifestation was available in 48 patients, among which the most common ones were arthritis (*n =* 37, 77.1%) and cutaneous lesions (*n =* 9, 18.8%). As for the clinical features, 13 cases were excluded since the clinical picture was described only at the time of diagnosis rather than the entire course (**Table 1**) (11). Of the remaining 63 patients, only 16 (25.4%) showed the classic triad (pyogenic arthritis, PG, and acne), while 23 (36.5%) had two major symptoms of the triad, and 24 (38.1%) had only one symptom. There were 54 (85.7%) patients who developed arthritis, usually involving the peripheral joints, including elbows, wrists, knees, ankles, hands, and feet, while axial joint involvement was occasionally seen (*n =* 3, 4.8%). Eleven (17.5%) patients displayed joint destruction leading to deformity and even underwent surgical interventions, such as joint replacement, synovectomy, and arthroplasty. Trauma or increased physical activity has been reported to induce arthritis flares (3, 12, 13). In regard to skin manifestations, 34 (54.0%) patients presented with acne and 26 (41.3%) with PG. Other skin lesions included pustular rash (*n =* 6, 9.5%), abscess (*n =* 5, 7.9%), pathergy reaction (*n =* 5, 7.9%), erythema (*n =* 5, 7.9%), skin ulcer (*n =* 2, 3.2%), psoriasis (*n =* 2, 3.2%), and rosacea (*n =* 1, 1.6%). Vaccinations and minor trauma have been reported to induce bruises or abscesses, which ulcerated and healed with scars (3, 4). Other clinical manifestations included fever (*n =* 17, 27.0%), osteomyelitis (*n =* 5, 7.9%), proteinuria (*n =* 5, 7.9%), inflammatory bowel disease (*n =* 4, 6.3%), splenomegaly (*n =* 4, 6.3%), otitis (*n =* 3, 4.8%), hidradenitis (*n =* 2, 3.2%), thrombosis (*n =* 2, 3.2%), uveitis (*n =* 1, 1.6%), granular T-lymphocyte clonal proliferation (*n =* 1, 1.6%), acute myelogenous leukemia (*n =* 1, 1.6%), and antiphospholipid syndrome (*n =* 1, 1.6%).

**Table 1:** Clinical features of patients with pyogenic arthritis, pyoderma gangrenosum, and acne syndrome.

Patients	Literature (*n* = 63)	Patient 1	Patient 2	Patient 3
Gender	M (53, 69.7%) F (23, 30.3%)^a^	F	F	M
Age at onset (years)	From infancy to 28 years	12	24	2
*PSTPIP1* variations	A230T, E250Q, E250K, E256G, V1408I, V408L, G403R, Q219H, D246N	Y119C	A230T	A230T
Initial symptom	Joint (37, 77.1%), skin (9, 18.8%), purulent otitis media (1, 2.1%)^b^	Skin	Joint	Joint
Acne	34 (54.0%)	No	Yes	No
PG	26 (41.3%)	Yes	Yes	No
Other skin lesions	Pustular rash (6, 9.5%), abscess (5, 7.9%), pathergy reaction (5, 7.9%), erythema (5, 7.9%), skin ulcer (2, 3.2%), psoriasis (2, 3.2%), rosacea (1, 1.6%)	Pustular rash	Pathergy reaction	No
Arthritis	54 (85.7%)	Yes	Yes	Yes
Triad completeness (%)	16 (25.4%)	–	Yes	–
Arthritis and pyoderma gangrenosum, PG (%)	7 (11.1%)	Yes	–	–
Arthritis and acne (%)	14 (22.2%)	–	–	–
PG and acne (%)	2 (3.2%)	–	–	–
Arthritis only (%)	19 (30.2%)	–	–	Yes
PG only (%)	3 (4.8%)	–	–	–
Acne only (%)	2 (3.2%)	–	–	–
Fever	17 (27.0%)	Yes	No	Yes
Other symptoms	Osteomyelitis (5, 7.9%), proteinuria (5, 7.9%), IBD (4, 6.3%), splenomegaly (4, 6.3%), otitis (3, 4.8%), hidradenitis (2, 3.2%), thrombosis (2, 3.2%), uveitis (1, 1.6%), TLGL (1, 1.6%), AML (1, 1.6%), APS (1, 1.6%)	Keratitis, recurrent abortion, urinary protein-positive, urinary occult blood 3+	Oral ulcer	Osteoporosis
Surgery history	11 (17.5%) (joint replacement, synovectomy, arthroplasty)	No	Synovectomy	Synovectomy

a n = 76.

b n = 48.

F, female; M, male; PG, pyoderma gangrenosum; IBD, inflammatory bowel disease; TLGL, granular T-lymphocyte clonal proliferation; AML, acute myelogenous leukemia; APS, antiphospholipid syndrome.

The documented gene variations for the 76 patients included A230T (*n =* 30, 39.5%), E250Q (*n =* 20, 26.3%), E250K (*n =* 5, 6.6%), E256G (*n =* 3, 3.9%), V1408I (*n =* 2, 2.6%), V408L (*n =* 1, 1.3%), G403R (*n =* 1, 1.3%), Q219H (*n =* 1, 1.3%), and D246N (*n =* 1, 1.3%). Eight putative cases of PAPA syndrome had proven negative for *PSTPIP1* coding or splicing mutations (1, 11, 13–15). Information on the genotype and phenotype of PAPA syndrome were documented and continuously updated in the Infevers database, a web-based resource (http://fmf.igh.cnrs.fr/ISSAID/infevers/). Among patients with A230T variation (*n =* 30), seven (23.3%) presented with the classic triad and 11 (39.3%) with PG. Among patients with E250Q variation (*n =* 20), two (10%) presented with the classic triad and five (25%) with PG. Of the five patients with E250K variation, the classic triad was observed in two (40%) and PG in all patients (100%). Due to the small number of cases, the phenotype–genotype relationship was not analyzed in other patients with rare variations.

Moreover, we summarized and analyzed the characteristics of patients with adult-onset PAPA syndrome (*n =* 6), which consisted of five cases from the literature (1, 14, 16–18) and patient 2. In terms of genotype, two patients (33.3%) had no gene mutations, while A230T was documented in three patients and G403R in one patient. In contrast, after excluding three cases where gene testing was not performed, only 14.0% (7/50) of childhood-onset patients did not have *PSTPIP1* mutations. Apart from patient 2 in our study (16.7%), no patients with adult-onset PAPA syndrome had a positive family history, while 11 (20.8%) patients with childhood-onset disease had a positive family history. All adult-onset PAPA syndrome patients (100%) had skin lesions, and 50% had arthritis. Of the 53 patients with the childhood-onset disease, 44 (83.0%) had skin manifestations, and 52 (98.1%) showed signs of arthritis.

Regarding treatment outcomes, 24 patients were treated with glucocorticoids, among whom six had poor response to either alone or combined with immunosuppressants or biological agents such as IL-1 antagonists or TNF-blocking agents, while a moderate to good response was observed in the remaining 18 patients. Sixteen patients were given combined immunosuppressants therapy, and a poor response was observed in six patients (37.5%). IL-1 antagonists were used in 21 patients, with only three (14.3%) ineffective. Furthermore, TNF-α antagonists were given to 10 patients, with two (20%) ineffective, and IL-6 antagonist to two patients with good response for arthritis (see **Table 2** for further details).

**Table 2:** Treatment and efficacy in patients with pyogenic arthritis, pyoderma gangrenosum, and acne syndrome.

Author	Case no.	Variants	Clinical characteristics	Poor response	Partial response	Good response
Guo, Y. N. (2)	1	c.36+68 G>A, c.137+47 G>C, and c.562+114C>G	Arthritis	MTX, CS		Tocilizumab
Sood, A. K. (19)	2	A230T	PG, acne, arthritis, pyogenic osteomyelitis, OM			Adalimumab + TAC
Sardana, K. (20)	3	E250Q	PG, cystic acne, arthritis			Dapsone + MTX
Maggio, M. C. (21)	4	V408L	Dermatitis, erythema, arthritis			Canakinumab
Martini, G. (22)	5	E250Q	Erythema, arthritis, osteonecrosis			CS
Loffler, W. (12)	6	A230T	Severe cystic acne, pustules, arthritis		Anakinra, canakinumab	
Kanameishi, S. (23)	7	E250K	PG, severe cystic acne, arthritis, proteinuria, splenomegaly, and resembling IBD	Etanercept	CS, adalimumab, anakinra, MTX, CsA	
Omenetti, A. (24)	8	E250Q	Arthritis		CS + MTX, anakinra	
	9	E250K	PG, hepatosplenomegaly, hypercalprotectinemia		Anakinra	Canakinumab
	10	E256G	PG, severe cystic acne, arthritis			Anakinra
	11	E256G	Arthritis			Anakinra
	12	E250Q	Arthritis			Anakinra + MTX
Zeeli, T. (16)	13	G403R	Severe acne, skin ulcerations, pustular rash, UC			Anakinra + CS
Lindwall, Elvira (25)	14	E250K	Acne, PG, arthritis, osteomyelitis	Infliximab, CS		
Park, B. M. (14)	15		PG, arthritis		CS	
Lambertucci, J. R. (26)	16		Nodulocystic acne, skin ulcers, arthritis, AML	MTX+CS		
Fathalla, B. M. (27)	17	Q219H, D246N	Abscess formation after immunization, arthritis			CS
Caorsi, R. (28)	18	E250Q	Arthritis, sterile osteolytic bone lesions	NSAIDs	CS	Anakinra
Demidowich, A. P. (29)	19	A230T	Sterile skin abscesses, PG, and cystic acne, arthritis, severe colonic inflammation	Plasmapheresis, THD, anakinra	TAC^a^	Infliximab
	20	E250Q	PG, sterile arthritis, sterile osteomyelitis, recurrent otitis	Etanercept	Anakinra^a^, infliximab^a^, CS^a^	Adalimumab
	21	A230T	Sterile abscesses, cystic acne, PG, sterile arthritis, recurrent otitis	IVIG, infliximab		MTX + adalimumab + CS
	22	A230T	PG, sterile arthritis			Anakinra
	23	E250K	PG, sterile arthritis, TLGL, vWF deficiency, thrombocytopenia, HA, pharyngeal papillomatosis	Anakinra	CsA^a^, TAC	
Lee, H. (30)	24	E250K	Multiple papulopustular lesions, PG, arthritis	CsA, CS		Adalimumab + CS
Schellevis, M. A. (18)	25	A230 T	Pustulosis, arthritis			CS
	26	A230 T	Acne, abscesses, arthritis, hidradenitis, APS			Anakinra
	27	A230 T	Acne, abscesses, arthritis, sarcoidosis, proteinuria,		CS	
Hong, Jin-Bon (15)	28		nodulocystic acne, PG, arthritis			CS
Brenner, M. (31)	29	A230T	PG, acne, erosive arthritis			Anakinra, CS
Tallon, B. (13)	30	E250Q	Rosacea, arthritis		NSAIDs, CS	
	31		PG, severe acne, arthritis, cervical ankylosis		NSAIDs, CS	
	32		Developing acne, arthritis			SASP + LEF
Dierselhuis, M. P. (32)	33	A230T	Mild acne, arthritis	CS		Anakinra
Cortis, E. (8)	34	A230T	Cystic acne, abscess, arthritis	CsA+CS		Etanercept
Carol A. Wise (33)	35	E250Q	PG	CsA, TAC, anakinra	CS	Infliximab
Lindor, Noralane M. (3)	36	A230T	Acne, PG, abscess after injection, arthritis, HS			AZA, CS, anakinra

a Discontinued due to side effects; partly responsive: chronic relapsing/not all symptoms could be controlled.

CS, glucocorticoids; UC, ulcerative colitis; MTX, methotrexate; OM, purulent otitis media; CsA, cyclosporine; CAV, cerebral arterial vasculopathy; NSAIDs, nonsteroidal anti-inflammatory drugs; HA, hemolytic anemia; TAC, tacrolimus; IVIG, intravenous immunoglobulin; AZA, azathioprine; LEF, leflunomide; SASP, sulphasalazine; THD, thalidomide; HS, suppurative hidradenitis.

## Discussion

So far, only one case of PAPA syndrome in the Chinese population has been documented in English literature (2). Herein we reported three adult patients with PAPA syndrome from two unrelated families in China. Our patients presented with the typical features of PAPA and *PSTPIP1* gene mutations; accordingly, a diagnosis of PAPA syndrome was made. This is, by far, the largest case series of PAPA syndrome from China to the best of our knowledge. Our study proved that the low incidence of PAPA syndrome in China might be due to poor awareness rather than nonexistence.

A literature review showed that arthritis was the common initial symptom of PAPA syndrome first observed from early childhood until puberty (34). It has been reported that joint symptoms tend to subside at puberty, with a concomitant increase in cutaneous symptoms (12, 29, 33). We also found that it usually took years to decades for these patients to be diagnosed. Delay in diagnosis may be accounted for by poor awareness of the disease and difficulties in distinguishing it from other diseases such as infectious and rheumatoid arthritis. In our study, patient 2 experienced disease onset during adulthood. By reviewing the literature, we found that adult-onset PAPA syndrome was more common in sporadic cases without genetic mutations and more likely to present with skin manifestations, while arthritis was only seen in nearly half of adult patients.

Nevertheless, patients with child-onset PAPA syndrome are more likely to have a family history of autoinflammatory diseases, and both arthritis and skin lesions are the cardinal features. Given the late onset of disease, atypical clinical presentation, and low disease prevalence, the diagnosis of adult-onset PAPA syndrome is a huge challenge for physicians. Taken together, we suggest that PAPA syndrome should be considered in patients presenting with recurrent arthritis, joint deformity, and spontaneous onset of rash, even in sporadic cases. Gene testing is critically helpful for diagnosis.

It is noteworthy that the clinical manifestations of PAPA syndrome are highly heterogeneous. Surprisingly, as many as 38.1% of patients presented with only one symptom of the classic triad during the disease course, emphasizing the importance of age at disease onset and family history in the differential diagnosis. Therefore, PAPA syndrome should be considered in children presenting with any of the components of the classical triad of symptoms (arthritis, PG, and acne). Additional manifestations have been reported, such as splenomegaly (23–25, 29) and inflammatory bowel disease (16, 17, 23). Moreover, the phenotypes of PAPA were variable even in patients carrying the same gene mutation. In our study, patients 2 and 3 were from the same pedigree and carried the same *PSTPIP1* mutation, yet the clinical manifestation and disease severity differed. These data indicated that the diversity of gene penetrance might alter the expression of PSTPIP1. It is worth mentioning that patients with E250K and A230T mutations were more likely to present with the classic triad of symptoms and high PG incidence. Accordingly, we highly suspected that E250K and A230T mutations in *PSTPIP1* were related to complete penetrance and PG. Nonetheless, more studies are needed to substantiate our findings.

Herein we identified a novel heterozygous *PSTPIP1* gene variant involving A>G transversion at cDNA position 356 (exon6) in patient 1. This variant was predicted to result in the substitution of tyrosine by a cysteine residue at amino acid position 119. According to the predictions by six different *in silico* analysis algorithms, this mutation will affect protein function: (1) Provean: deleterious (http://provean.jcvi.org/index.php; a score of 0.38), (2) SIFT: damaging (http://sift.jcvi.org/www/SIFT_enst_submit.html; a score of 0.045), (3) Polyphen2-HDIV: probably damaging (http://genetics.bwh.harvard.edu/pph2/; a score of 0.983), (4) polyphen2-HVAR: possibly damaging (http://genetics.bwh.harvard.edu/pph2/; a score of 0.905), (5) Mutation Taster: disease-causing (http://www.mutationtaster.org/; a score of 1), and (6) M-CAP: damaging (http://bejerano.stanford.edu/mcap/; a score of 0.044). However, further functional studies are needed to clarify the pathogenic role of this novel *PSTPIP1* variant. Intriguingly, in the present study, patient 1 also presented with keratitis, which has not been previously documented in patients with PAPA syndrome, in contrast to uveitis that has been occasionally reported (13). Moreover, the incidence of hematuria and proteinuria in patient 1 was consistent with the literature, with previous reports of proteinuria in five patients with PAPA syndrome (3, 17, 18, 35). Therefore, we speculate that other manifestations beyond the cardinal features of PAPA syndrome should be further concerned.

Given that recurrent episodes of destructive inflammation of the joints can lead to joint deformity and the risk of infection and extensive ulcers secondary to PG, early diagnosis and prompt treatment are required to avoid severe complications. Generally speaking, the arthritis episodes in this patient population are responsive to glucocorticoids. Compared to low- or medium-dose intraarticular injections of corticosteroids followed by short-term oral treatment, high-dose intraarticular injections of corticosteroids within the first days are recommended to treat an arthritis flare and avoid or minimize acne flares and erosive joint disease (12). However, complete remission was achieved with glucocorticoids alone in only a few cases, which reminded us of the critical importance of the quest for new treatments. Disease-modifying or immunosuppressive agents have been reported to be insufficient for the control of disease activity. In addition, a few successful experiences can be achieved from the literature. Sood AK et al. reported the effective treatment of PAPA syndrome with dual adalimumab and tacrolimus therapy (19). Another study demonstrated that IL-1 blocking agents alone worked well for arthritis in most patients, and the addition of isotretinoin to biotherapies was required to control acne in several patients (16). Moreover, Demidowich et al. noticed that TNF-α blockade appeared to be effective in treating the cutaneous manifestations of PAPA syndrome, and responses to TNF and Il-1 blocking agents were incomplete in the vast majority of patients, suggesting the contribution of additional pathogenetic mechanisms (29). Jesus et al. also hypothesized that a partial response to IL-1 inhibition suggested the involvement of additional cytokine pathways (36). Laberko A et al. reported five PAMI patients with *PSTPIP1* E250K who underwent a successful hematopoietic stem cell transplantation (37). However, bone marrow transplantation has not been reported in PAPA patients, to the best of our knowledge. Increasing the recognition of the peculiarities of gene expression in PAPA syndrome and further elucidation of its pathogenesis will aid in developing new treatment approaches in the future.

## Data Availability Statement

The original contributions presented in the study are included in the article/Supplementary Material. Further inquiries can be directed to the corresponding authors.

## Ethics Statement

This research was approved by the Institutional Review Board of Peking Union Medical College Hospital and performed according to the Declaration of Helsinki. Written informed consents were obtained from all participants.

## Author Contributions

All authors listed have made a substantial, direct, and intellectual contribution to this work and approved it for publication.

## Funding

This work was supported by the Natural Science Foundation of Beijing (grant no. 7192170), the Chinese Academy of Medical Sciences Innovation Fund for Medical Sciences (grant no. 2017-I2M-3-001), the Peking Union Medical College Hospital Foundation for Distinguished Young Scholars (grant no. JQ201705), and the National Key Research and Development Program of China (grant nos. 2016YFC0901500 and 2016YFC0901501).

## Conflict of Interest

The authors declare that the research was conducted in the absence of any commercial or financial relationships that could be construed as a potential conflict of interest.

## Publisher’s Note

All claims expressed in this article are solely those of the authors and do not necessarily represent those of their affiliated organizations, or those of the publisher, the editors and the reviewers. Any product that may be evaluated in this article, or claim that may be made by its manufacturer, is not guaranteed or endorsed by the publisher.
